# Best abstracts from the APAGBI Annual Scientific Meeting 2024

**DOI:** 10.1111/pan.15039

**Published:** 2024-11-25

**Correspondence**

Johnny Kenth, The Royal Manchester Children's Hospital

Email: johnny.kenth@mft.nhs.uk

As chair of the scientific committee and representative of the organising committee for the Association of Paediatric Anaesthetists of Great Britain and Ireland.

## List of Authors (lead author for each abstract)

1. Wensley, Frances; University Hospital London NHS Foundation Trust, NIHR Southampton Biomedical Research Centre, Southampton, UK
2. Addy, Melissa; Great Ormond Street Hospital, London, UK
3. Jenkins, Victoria; The Royal London Hospital, London, UK
4. Lyne, Tom; Barnet Hospital, Royal Free London NHS Foundation Trust, Barnet, UK
5. Keough, Jamie; Alder Hey Children's Hospital, Liverpool, UK
6. Burton, Zoe A.; Sheffield Children's NHS Foundation Trust, UK
7. Kenth, Johnny J.; The Royal Manchester Children's Hospital, UK
8. Riley, Catherine; Sheffield Children's Hospital, Sheffield, UK
9. Arnold, Phillip; Alder Hey Children's Hospital, Liverpool, UK

The 2024 Annual Scientific Meeting of the Association of Paediatric Anaesthetists (APAGBI) was held in Newcastle. From 107 submissions, the top‐scoring abstracts were selected for oral presentation, representing a high calibre of presentations that included original primary research, systematic reviews, quality improvement initiatives, and education projects. These presentations showcased a diverse range of important paediatric anaesthesia advancements.

The Pediatric Anesthesia journal prize for the best overall abstract was awarded to Dr. Wensley for the study, *“Perioperative Paracetamol Prescribing in Obese and Overweight Children: Secondary Analysis of the PEACHY Study”*. Additional prizes were awarded for the best quality improvement project to Dr. Addy for the project *“Implementing an Enhanced Recovery Programme for Adolescent Idiopathic Scoliosis Surgery”* and for the best education project to Dr. Jenkins for *“Paediatric Regional Anaesthesia Scanning Club at The Royal London Hospital”*. The Best Poster Presentation was awarded to Dr. Lyne for the paper titled "Risk of Dying in Paediatric Anaesthesia: Evaluation, Discussion and Consent."

## Perioperative paracetamol prescribing in obese and overweight children: Secondary analysis of the perioperative childhood obesity (PEACHY) Study

### F. L. Wensley ^1^; S. Heikal^2^; H. Lewis^3^; T. Bennett^4^; M. R. Edwards^1^; Z. A. Burton^5^; Paediatric Anaesthesia Trainee Research Network (PATRN)^6^

#### ^1^University Hospital London NHS Foundation Trust, NIHR Southampton Biomedical Research Centre, Southampton, UK; ^2^Elisabeth Twee‐Steden Zeikenhuis, Tilburg, The Netherlands; ^3^Evelina Children's Hospital, London, UK; ^4^University Hospital London NHS Foundation Trust, Southampton, UK; ^5^Sheffield Children's NHS Foundation Trust, UK; ^6^Paediatric Anaesthesia Trainee Research Network, London, UK

**Introduction and Aims:** Paracetamol is the most frequently administered analgesic agent for children undergoing surgery in the UK.^1^ Prescribing is traditionally based on total body weight (TBW) or age‐banded dosing. However, there is limited evidence on optimal dose adjustments for children living with overweight and obesity (OAO); a cohort comprising almost a quarter of the UK paediatric surgical population.^2^ Severely obese children frequently have underlying cardiometabolic dysfunction, and metabolic associated fatty liver disease may increase susceptibility to paracetamol hepatotoxicity.^3^ Lack of drug‐specific pharmacokinetic data and expert guidance for this patient group, has left individual prescribers to determine dose adjustments. We aimed to describe and understand paracetamol prescribing practices in UK children with OAO.

**Methods:** PEACHY study methodology has been published previously.^2^ All paediatric patients (2 to 15 years) undergoing elective, day‐case, or emergency procedures under general anaesthesia were eligible for inclusion. Patient demographics, surgical procedure, height, weight, paracetamol prescribed, intraoperative and postoperative care were documented. Adjusted body weight (AdjBW) was calculated according to recent clinical guidance.^4^ Data were excluded for not meeting age criteria (<2 or ≥16 years), insufficient information to calculate body mass index (BMI), or immediate postoperative admission to PICU (no PACU data). Patients not prescribed paracetamol perioperatively, or those appropriately prescribed a maximal 1g dose, were also excluded (due to risk of high BMI adolescents skewing mean data). In accordance with the National Institute for Health and Care Excellence5 recommendations; the British 1990 child growth reference (UK90) was used to assign age and sex‐specific BMI centiles.6 “Clinical” BMI centile thresholds were used to define children as underweight, healthy, overweight, obese (>98th centile), or severely obese (>99.6th centile, a subset of “obese”).

We analysed perioperative paracetamol prescriptions comparing TBW and AdjBW doses across 102 UK sites. Paracetamol dose was almost normally distributed and thus expressed as mean (standard deviation, SD). In smaller subgroups where this was not the case, median (interquartile range, IQR) was used. Two‐sample *t*‐tests or Mann–Whitney tests were used to evaluate differences between means or medians, chi‐squared tests for correlation between categorical variables, and ANOVA or Kruskal–Wallis tests for comparison of categorical and quantitative variables. To quantify whether paracetamol doses varied across BMI categories and relevant covariates, an interaction term was included in ANOVA analyses between each covariate and BMI. Data were analysed using STATA version 16.1 (StataCorp LLC).

**Results:** 3275 patients were included in the current analyses. Of these, 20.2% (662) were living with OAO. There was significant variation in dose across BMI categories; patients with OAO received lower doses per kilogram of TBW (mean [SD] 14.83 [2.45], 13.88 [2.45] and 12.76 [3.55] mg.kg‐1 respectively) compared to normal or underweight patients (15.83 [2.84] and 15.49 [2.50] mg.kg‐1 respectively, *p*‐value for ANOVA < 0.001). Paracetamol dose varied significantly across sites. Mean AdjBW doses for patients with OAO were 16.91 (2.79), 16.81 (2.92), and 17.11 (4.64) mg.kg‐1, respectively.

**Discussion and Conclusion:** This is the first national, prospective, multi‐centre study reporting on perioperative paracetamol prescribing amongst UK children. There is a wide range in paracetamol dose prescribed for children with OAO. However, this study provides evidence that anaesthetists are attempting to adjust for excess body weight in this population, using an estimate between AdjBW and TBW. This work supports recent guidelines,^4,7^ which suggest AdjBW dose may be the safest perioperative approach to avoid risks associated with overdose in this potentially vulnerable population.

**References**

1. Star K, Choonara I. How safe is paracetamol? *Archives of Disease in Childhood* 2015;100:73‐74.
2. Burton Z, Lewis R, Bennett T et al. Prevalence of PErioperAtive CHildhood obesitY in children undergoing general anaesthesia in the UK: a prospective, multicentre, observational cohort study. *British Journal of Anaesthesia*. 2021; 127 (6): 953e961.
3. Skinner AC, Perrin EM, Moss LA, Skelton JA. Cardiometabolic Risks and Severity of Obesity in Children and Young Adults. *N Engl J Med*. 2015 Oct;373(14):1307‐17.
4. Burton Z, Russell K, Saddington F et al. Anesthesia for Children living with Obesity. Single Sheet Guideline. https://www.sobauk.co.uk/guidelines‐1 (Last accessed 11th November 2023).
5. National Institute for Health and Care Excellence. Obesity: identification, assessmentand management. clinical guideline CG189 2014. https://www.nice.org.uk/guidance/cg189. (Last accessed 4th November 2023).
6. Cole TJ. Growth monitoring with the British 1990 growth reference. *Archives of Disease in Childhood*. 1997;76:47‐49.
7. UK Medicines Information (UKMi) pharmacists and Neonatal and Paediatric Pharmacists Group (NPPG) How should medicines be dosed in children who are obese? 2021. https://www.sps.nhs.uk/articles/how‐should‐medicines‐be‐dosed‐in‐children‐who‐are‐obese/. Last accessed 03/12/2023.

## Implementing an enhanced recovery programme for adolescent idiopathic scoliosis surgery

### M. Addy; E. Broomfield; T. J. W. Dawes; M. George

#### Great Ormond Street Hospital, London, UK

**Background:** Recovery from posterior spinal fusion (PSF) for adolescent idiopathic scoliosis (AIS) can be slowed by pain, gastrointestinal symptoms, and delayed mobilisation.

Enhanced recovery programmes (ERP) for this patient group are heterogeneous^1^ but may reduce the length of stay without increasing complications^2,3^.

**Problem:** There was no standardised peri‐operative care pathway for patients with AIS undergoing PSF at our institution. Our seven‐day planned admission was longer than reported elsewhere^2,3^.

We aimed to improve functional recovery by reducing opioid‐related side effects and accelerating mobilisation.

**Strategy for Change:** Thirty consecutive cases were retrospectively reviewed to define the pre‐intervention peri‐operative management and outcomes. Patient anthropometrics (age, sex, weight, ASA status), surgical characteristics (instrumented levels, operative duration, post‐operative drop in serum haemoglobin), and anaesthetic management were recorded. Post‐operative trajectory was defined by analgesia use, pain scores, gastrointestinal symptoms (nausea, vomiting, constipation (BNO > 3 days)), and time to enteral intake, urinary catheter removal, mobility outcomes, and hospital discharge. A ward‐based ERP was developed in conjunction with the multidisciplinary team focusing on patient and family education and expectation management, multi‐modal analgesia regimes and post‐operative protocolised daily targets for all aspects of care. The first fifty consecutive patients post implementation were followed prospectively with two interim safety reviews.

Data were stored securely on Microsoft Excel, analysed using GraphPad Prism, and are reported as mean (SD) for normal and median (IQR) for skewed data. Data were compared using unpaired *t*‐tests and Mann–Whitney tests respectively.

**Measure of Improvement:** The pre‐ERP and ERP groups were comparable for patient and surgical characteristics except for longer operative time in the ERP group (305 (52) vs 349 (64) minutes, *p* = 0.002).

Hospital length of stay was reduced by one day in the ERP group (5 (5‐6) vs 6 (6‐7) days, *p*<0.001). All other measured timepoints were also reduced including urinary catheter removal (1 (1‐1) vs 3 (2‐4) days, *p*<0.001) and physiotherapy discharge (3 (3‐4) vs 4 (3‐5) days, *p* < 0.001). In the ERP group, parenteral morphine was used for a shorter time, with a lower total dose (1.1 (0.9‐1.4) vs 1.8 (1.5‐2.3) mg.kg‐1, *p* < 0.001). Pain scores were similar but with less nausea (54 vs 93%), vomiting (64 vs 73%) and constipation (22 vs 60%). Compliance with the ERP interventions was higher for the anaesthetic protocol and post‐operative prescribing for days 0‐1 (both 89%) than ward‐based prescribing from day 2 onwards (43%).

**Lessons Learnt:** Enhanced recovery is feasible in this patient group, improving functional recovery and reducing the length of stay. Compliance with the post‐operative medication regime fell from day two. An electronic prescribing bundle to facilitate the prescription of the full regime on day 0 is being developed.

**Message for Others:** The Spinal Clinical Nurse Specialists have taken ownership of the ERP, which has facilitated ongoing staff engagement and education.

**References**

1. Gadiya AD, Koch JEJ, Patel MS, Shafafy M, Grevitt MP, Quraishi NA. Enhanced recovery after surgery (ERAS) in adolescent idiopathic scoliosis (AIS): a meta‐analysis and systematic review. Spine Deform. 2021;9(4):893‐904.
2. Koucheki R, Koyle M, Ibrahim GM, Nallet J, Lebel DE. Comparison of interventions and outcomes of enhanced recovery after surgery: a systematic review and meta‐analysis of 2456 adolescent idiopathic scoliosis cases. Eur Spine J. 2021 Dec;30(12):3457‐3472.
3. Subramanyam R, Muhly WT, Goobie SM. Enhanced recovery: The evolution of pediatric spinal fusion care. Pediatr Anesth. 2020;30(10):1066‐1067.

## Paediatric regional anaesthesia scanning club at the Royal London Hospital

### V. Jenkins; R. Carrington; M. Roberts

#### The Royal London Hospital, London, UK

**Introduction and Aims:** Regional anaesthesia is an exciting and rapidly developing branch of our specialty. It conveys numerous benefits in the paediatric population including improved early postoperative pain scores and a reduction in opioid analgesia^1^. This, combined with evidence of a low incidence of complications make regional anaesthesia a fundamental part of modern paediatric anaesthetic practice^2^. A structured educational programme is essential to upskill our department and incorporate regional anaesthesia into our everyday practice. Inspired by the release of the Paediatric Plan A Blocks from RA‐UK^3^ we initiated a Paediatric Scanning Club for both consultants and trainees to attend.

We aimed to provide:

1. A concise weekly educational session to cover block indications and relevant anatomy.
2. An opportunity to increase hands‐on ultrasound scanning experience and identification of the appropriate sonoanatomy.
3. An opportunity for discussion of differences between the paediatric and adult block, including dosing of local anaesthetic.

**Methods:** In 2023, we designed and implemented a 7‐week programme covering common blocks used in paediatrics, including femoral, popliteal, axillary, and rectus sheath blocks.

Each session was led by a Stage 2+ trainee on their paediatric anaesthesia placement and supervised by a paediatric consultant anaesthetist proficient in the block of the week.

The trainee leading the session prepared a short presentation including the indications, anatomy, patient positioning, sonoanatomy, recommended local anaesthetic dose, and a short video of the block being performed. Following this, there was an opportunity to practice identifying the sonoanatomy on volunteers, and practice needling using jelly containing hidden targets. A survey was distributed to collect feedback after the first 7‐week programme. Three 7‐week programs have now been conducted.

**Results:** Responses were received from both trainees (67%) and consultants (33%) with 75% of total trainees on the rotation completing the survey (6/8).

- 100% of respondents thought that the Scanning Club sessions they attended improved their theoretical knowledge of the relevant block in paediatric patients.
- For every block covered, following the attendance of the scanning club session, more attendees felt confident to perform that paediatric block independently (with a supervisor in the room).
- Suggestions for improvement included: Initiating a changeover time halfway through the session for consultants and trainees who were paired up in theatres to swap over allowing both to attend, and exploring the possibility of scanning practice on paediatric volunteers.

**Discussion:** This simple format trainee‐led teaching session has been positively received by both consultants and trainees and could be easily replicated in other hospitals. The programme has now been repeated three times at RLH, following the three‐monthly trainee rotation, with the organisers acting on feedback to make improvements each time. Specific aspects that people have consistently enjoyed include the “small group, focused teaching and regular practice” and that it is a “great opportunity to revise common blocks used in paediatric anaesthesia”.

**References**

1. P.‐A. Lönnqvist, N. S. Morton, Postoperative analgesia in infants and children, BJA: British Journal of Anaesthesia, Volume 95, Issue 1, July 2005, Pages 59–68.
2. Polaner DM, Taenzer AH, Walker BJ, Bosenberg A, Krane EJ, Suresh S, Wolf C, Martin LD. Pediatric Regional Anesthesia Network (PRAN): a multi‐institutional study of the use and incidence of complications of pediatric regional anesthesia. Anesthesia & Analgesia. Dec 2012 Dec;115(6):1353‐64.
3. RA‐UK 2023, Paeds Plan A Posters, https://ra‐uk.org/index.php/plan‐a‐blocks‐home/plan‐a‐paeds/pads‐plan‐a‐posters.html

## Decision‐making and multidisciplinary team approach for complex airway management in Morquio A Syndrome: Experience from a National Referral Service

### J. J. Kenth ^1^; E. Maughan^2^; S. Wilkinson^1^; M. de Kruijf^1^; S. Jones^1^; C. Butler^2^; I. A. Bruce^1^; R. Hewitt^2^; R. Nandi^2^

#### ^1^The Royal Manchester Children's Hospital, UK; ^2^Great Ormond Street Hospital for Children, London, UK

**Background/Context:** Mucopolysaccharidosis type‐IVA (MPS IVA or Morquio A Syndrome) is a rare lysosomal storage disease associated with progressive, multi‐level central airway obstruction due to tracheomalacia, stenosis, and tortuosity. In severe phenotypes, disease progression culminates in near‐fatal, airway obstructions, which is the leading cause of death^1,2^. Traditional management strategies have been palliative, focusing on symptom relief through non‐invasive ventilation (NIV). We aimed to explore the critical role of multidisciplinary team (MDT) decision‐making for a novel surgical approach (tracheal resection combined with partial upper manubriectomy) to ameliorate critical airway obstruction in children with MPS IVA^3,4^.

**Problem:** Patients who progress to critical airway obstruction despite enzyme replacement therapy (ERT) lack definitive treatment options, with palliative care being the primary approach. This gap necessitates a novel surgical solution to manage the profound multi‐level airway disease and improve quality of life.

**Strategy for Change:** In a collaborative effort to address near‐fatal airway obstruction in Morquio Syndrome, Royal Manchester Children's Hospital (RMCH), a leading European centre for metabolic disorders, partnered with Great Ormond Street Hospital (GOSH), home to the national tracheal service. An MDT approach was initiated to develop a referral‐to‐surgery pathway. This involved setting up a framework for pre‐procedural planning, patient selection, and decision‐making, leveraging the combined expertise of RMCH and GOSH. The strategy sought to refine the identification of surgical candidates and streamline the process from referral to surgery, optimising the timing for intervention

**Measure of Improvement:** The effectiveness of the new pathway was evaluated by measuring the referral‐to‐surgery time (4‐6 months), postoperative improvements in respiratory function through spirometry, and enhanced quality of life (QoL)via PedsQL (v4) questionnaires^5^. All seven patients showed significant postoperative improvements in respiratory function, with a mean increase in FEV1 of 0.68 litres (95% CI: 0.45‐0.91; SD: 0.20). Postoperatively, all but one patient were able to discontinue non‐invasive ventilation, with the remaining patient significantly reducing NIV support. QoL assessments indicated substantial improvements across multiple domains, with mean PedsQL scores increasing from 51.2 preoperatively to 78.4 postoperatively, reflecting a 53% improvement. The MDT approach also improved the consistency of decision‐making and communication with referring centres and patients.

**Lessons Learnt:** The initiative demonstrated the critical importance of centralised expertise and the benefits of a collaborative MDT approach. The robust referral pathway facilitated the surgical management of nine patients across the UK, with significant improvements noted postoperatively, validating the effectiveness of the strategy.

**Message for Others:** The centralised approach and shared decision‐making serve as a model for the management of rare diseases requiring highly specialised care. This QI project illustrates how pooling resources and knowledge within a national framework can lead to improved patient outcomes and serve as a guide for similar high‐risk interventions in other rare diseases.

**Conclusion:** This QI initiative underscores the value of a national referral pathway and MDT approach in managing complex airway disease in Morquio A Syndrome. The collaborative efforts between RMCH and GOSH have not only improved patient outcomes but have also set the groundwork for the management of complex medical conditions, promoting a model that can be replicated for other rare diseases.

**References**

1. Broomfield A, Kenth J, Bruce IA, Tan HL, Wilkinson S. Respiratory complications of metabolic disease in the paediatric population: A review of presentation, diagnosis and therapeutic options. Paediatric Respiratory Reviews. 2019 Nov;32:55–65.
2. Kenth JJ, Thompson G, Fullwood C, Wilkinson S, Jones S, Bruce IA. The characterisation of pulmonary function in patients with mucopolysaccharidoses IVA: A longitudinal analysis. Mol Genet Metab Rep. 2019 Sep;20:100487.
3. Frauenfelder C, Maughan E, Kenth J, Nandi R, Jones S, Walker R, et al. Tracheal Resection for Critical Airway Obstruction in Morquio A Syndrome. Case Reports in Pediatrics. 2023 May 3;2023:e7976780.
4. Kenth, J., Maughan, E., Butler, C. R., Gabriel, J., Rouhani, M., et al. (2024). Novel approach for tracheal resection in Morquio A syndrome with end‐stage critical airway obstruction: A UK case series. Orphanet Journal of Rare Diseases, 19(274). https://doi.org/10.1186/s13023‐024‐03253‐3.
5. Varni, J.W., Seid, M., Knight, T.S., Uzark, K., & Szer, I.S. (2002). The PedsQL 4.0 Generic Core Scales: Sensitivity, responsiveness, and impact on clinical decision‐making. *Journal of Behavioral Medicine, 25*(2), 175‐193.

## Improving outcomes in unilateral pelvic osteotomy in children and young people: The impact of continuous Quadratus Lumborum catheter technique in an epidural‐led service

### J. Keough; E. Robinson; I. Okonkwo; S. Roberts; E. Kehoe

#### Alder Hey Children's Hospital, Liverpool, UK

**Background:** At Alder Hey Children's Hospital, lumbar epidural (LE) has been the gold standard regional technique for unilateral pelvic and proximal femoral osteotomy (UPAPFO). These patients are discharged to Level 1+ ward beds and have high nursing requirements. Complications such as hypotension, motor blockade, and urinary retention make LEs a less desirable option^1^, particularly in patient groups with frailty. LE use can also be limited by coagulopathy, previous spinal surgery, etc^2^. Quadratus lumborum catheters (QLC) are a peripherally inserted alternative to LE in patients undergoing UPAPFO, they require fewer nursing interventions and may avoid a number of the challenges associated with LE.

**Problem:** There is a paucity of paediatric data, but adult literature suggests that quadratus lumborum blocks confer reduced opioid consumption, pain score, length of stay (LOS), and other complications (hypotension, motor weakness, retention etc.) in hip arthroplasty and arthroscopy^3,4^. QLC might therefore benefit our UPAPFO patients.

**Strategy:** We used the Plan‐Do‐Study‐Act model to embed QLC use into routine practice alongside a multimodal analgesic regimen in patients undergoing UPAPFO. We delivered a departmental training programme for QLB and QLC including lectures, practical workshops, and real‐time clinical support. Patient outcome data and clinical experiences of the acute pain team were presented to the department. Recommendations are ongoing.

**Measure of Improvement:** Between January 2021 and July 2023, 48 patients aged 1‐17 years underwent UPAPFO utilising LE (*n* = 31(65%)) or QL catheter (*n* = 17(35%)). QLC and LE patients had similar demographics (*p* > 0.5: age, gender, weight, ASA). Anaesthetic, perioperative analgesia, and outcome data (pain scores, complications, and LOS) were recorded. Audit ID. 6679. Data analysis was performed utilising GraphPad Prism version 10.2.3 for macOS (San Diego, CA, USA)

**Learning:** We ascertained that QLC combined with multimodal analgesia conferred a non‐inferior quality of comfort and similar levels of safety when compared to LE in our patients. Total pain scores were similar in QLC and LE groups on day 1: Median (IQR) = 6 (1.5 ‐ 16.5) for QLC vs 6 (3.0 ‐ 17.0) for LE; *p* > 0.05; 95% CI = ‐5.0 to 5.0 and day 2: Median (IQR) = 7 (1.5 ‐ 20.0) for QLC vs 11 (2.0 ‐ 21.0) for LE; *p* > 0.05; 95%CI = ‐5.0 to 9.0. Numerically fewer QLC patients experienced complications; 5.8% for QLC vs 32.6% for LE; *p* > 0.05; 95% CI = 0.01 to 1.07. One child was converted from LE to QLC following a dural tap during LE placement. There was also a trend towards a shorter LOS in QLC patients. Median (IQR) = 5 (4.0‐6.0) for QLC days vs 6 (5.0 ‐ 8.0) for LE days; *p* = 0.0256; 95% CI = 0.0 to 2.0).

**Ongoing Outcomes:** Locally we have agreed upon a UPAPFO pathway with QLC forming the primary mode of analgesia, this now runs alongside our existing LE pathways. We are in discussion with other centres to expand our local database to a multicentre regional/national database and are collaborating within our multidisciplinary team to improve the quality of recorded outcome measures e.g. physiotherapy milestones. We are currently also evaluating the effectiveness of single shot quadratus lumborum blocks in simpler osteotomy groups e.g. unilateral Salter's osteotomy with a view to further improving the quality of care and quality of patient and parental experience, alongside reducing length of stay. Our hope is to engage in a randomised control trial looking at QLC in UPAPFO.

**Message:** QLC presents an alternative and effective analgesic option in children and young people undergoing UPAPFO with comparable analgesia to LE and possibly reduced complications. Further rigorous studies are needed to validate the efficacy and safety of this novel technique to corroborate our preliminary findings.

**References**

1. Walker B, Long J. Complications in Pediatric Regional Anesthesia: An Analysis of More than 100,000 Blocks from the Pediatric Regional Anesthesia Network. Anesthesiology. 2018;129(1):721‐732.
2. Cook TM, Counsell D, Wildsmith JA. Major complications of central neuraxial block: report on the Third National Audit Project of the Royal College of Anaesthetists (NAP3). BJA. 2009;102(2):179‐190.
3. Huda A, Minhas R. Quadratus Lumborum Block Reduces Postoperative Pain Scores and Opioids Consumption in Total Hip Arthroplasty: A Meta‐Analysis. Cureus. 2022;14(2):e22287.
4. Blackwell RE, Kushelev M. A Comparative Analysis of the Quadratus Lumborum Block Versus Femoral Nerve and Fascia Iliaca Blocks in Hip Arthroscopy. Arthrosc Sports Med Rehabil. 2020;3(1):7‐13.

## Can an anaesthetic preoperative excess weight pathway improve outcomes for children living with severe obesity?

### Z. A. Burton; N. Aswani; L. Cronin; J. Reynolds; N. Marshall; V. Hodgetts

#### Sheffield Children's NHS Foundation Trust, UK

**Background:** In 2019, it was found that 1 in 4 UK children undergoing general anaesthesia were living with overweight and obesity (OAO)^1^. The prevalence was further exacerbated by the effects of the COVID‐19 pandemic. Childhood obesity leads to a five‐fold increased likelihood of adulthood obesity, associated chronic conditions and reduced life expectancy^2^. The current estimated UK cost of OAO is £98 billion. Lifestyle interventions can help reduce this burden.

**Problem:** Children with OAO undergo more surgical procedures have increased anaesthetic risk and poorer perioperative outcomes. Nationally, there is no robust preoperative screening process to risk‐assess or consent for obesity related outcomes, representing a missed opportunity for both risk reduction and health promotion^1^. Identifying OAO preoperatively permits screening for comorbidities that may affect the perioperative course, and allows appropriate consent for increased anaesthetic risk ahead of the day of surgery. Children and their families can then be given an opportunity to make lifestyle interventions within a supportive environment, with the aim of reducing their perioperative risk.

**Strategy for Change:** In March 2021 a weekly preoperative excess weight clinic was initiated at Sheffield Children's Hospital for severely obese children (>99.6th BMI centile), involving face‐to‐face appointments with an anaesthetic consultant trained in motivational interviewing. Anaesthetic risk and comorbidities were evaluated, with individualised age‐appropriate lifestyle goals agreed upon, and tailored to biopsychosocial needs. Appropriate referrals were made to paediatricians, Tier 2 weight management programmes, and an exercise and physical activity therapist. Since 2021, major iterative improvements included relocation to the Advanced Wellbeing Research Centre with gym access, establishing a Sleep Nurse Specialist referral pathway, and fostering close collaborative working and referral to Tier 3 clinics. Additionally, an app‐based digital patient information repository was introduced, along with national guidance on consent and risk discussions^3^. Other improvements included Nurse Practitioner‐led excess weight clinics and a combined oral health promotion clinic.

**Measure of Improvement:** The natural trajectory for severely obese children is to continue gaining weight into adulthood. Significant improvement in body composition and cardiometabolic risk have been observed following reductions in BMI z‐score more than 0.25, with even greater benefits seen with reductions of more than 0.5 (4). The clinic database included 304 children (41.1% female; mean age 10.4 years, SD 4.01), of which 173 (56.9%) had undergone surgery. 72.3% (*n* = 125) reduced their BMI z‐score preoperatively (mean ‐0.28; SD 0.26); 27.7% (*n* = 48) and 13.3% (*n* = 23) reduced their BMI z‐score by >0.25 and >0.5 respectively. In several cases, obesity reduced sufficiently to fall at least one weight category (22.0%, *n* = 38), no longer be classified “severely obese” (12.7%, *n* = 22), demonstrate reversal of comorbidities (insulin resistance, liver dysfunction), no longer require CPAP for OSA, or post‐operative HDU beds.

**Lessons Learned:** This retrospective study provides evidence that lifestyle interventions in the preoperative period can be sufficient to exert a positive effect upon cardiometabolic risk. Outcomes from lifestyle intervention in this setting have superseded expectations and one possibility is that families facing imminent increased perioperative risks may be more motivated to adopt positive behavioural change. Over half of children in this study were amongst the lowest two deciles of socioeconomic deprivation, reflecting the strong link with childhood obesity. Families valued being listened to in a non‐judgemental face‐to‐face setting with an individualised multidisciplinary approach to lifestyle changes.

**Message for Others:** As health professionals, everyone has a role in health promotion and ensuring sustainability. This initiative has shown that by capitalising on this “teachable moment”, significant perioperative risk modification and health benefits are feasible, with potential for longer term health and socioeconomic effects.

**References**

1. Burton ZA, Lewis R, Bennett T, McLernon DJ; Paediatric Anaesthesia Trainee Research Network; Engelhardt T, Brooks PB, Edwards MR. Prevalence of PErioperAtive CHildhood obesitY in children undergoing general anaesthesia in the UK: a prospective, multicentre, observational cohort study. *Br J Anaesth*. 2021;127(6):953‐961.
2. Simmonds M, Llewellyn A, Owen CG, Woolacott N. Predicting adult obesity from childhood obesity: a systematic review and meta‐analysis. *Obes Rev*. 2016;17(2):95‐107.
3. Burton ZA, Saddington F, Russell K and the Society for Obesity and Bariatric Anaesthesia, 2024. Anaesthesia consent for children and young people living with obesity. https://www.sobauk.co.uk/guidelines‐1 (Accessed 7th August 2024)
4. Reinehr T, Lass N, Toschke C, Rothermel J, Lanzinger S, Holl RW. Which amount of BMI‐SDS reduction is necessary to improve cardiovascular risk factors in overweight children? *J Clin Endocrinol Metab* 2016;101(8):3171‐9.

## PINEAPPLE: PaedIatric caNcellation ratEs And PerioPerative clinicaL Evaluation

### C. L. Riley^1^; T. Bennett^2^; H. Lewis^3^

#### ^1^Sheffield Children's Hospital, Sheffield, UK; ^2^University Hospital Southampton, UK; ^3^Evelina Children's Hospital, London, UK

**Introduction:** Paediatric pre‐operative assessment (P‐POA) is an emerging field with the potential to improve outcomes from paediatric surgery. Best practice P‐POA standards are published (1) and it is recommended that “children undergoing anaesthesia should be offered a preadmission pre‐assessment service” (2). PINEAPPLE is a multicentre, prospective observational cohort study. The primary aims of this study were to establish the proportion of children 16 years old and under seen in P‐POA clinic prior to an elective procedure under general anaesthetic (GA) and to establish the format of that pre‐assessment. The secondary aims included establishing the on‐the‐day cancellation rate for children presenting for surgery and understanding the incidence and impact of anxiety.

**Methods:** Invitations were sent to members of the Paediatric Anaesthetic Trainee Research Network (PATRN) and the Association of Paediatric Anaesthetists of Great Britain and Ireland (APAGBI). A total of 98 hospitals enrolled and registered the study with their local governance team. The HRA toolkit determined that this study is a nationally coordinated, locally conducted audit, hence ethical approval and patient consent were not required.

**Data Collection:** Recruitment occurred prospectively between April and June 2023 over a 14‐day period. All patients ≤ 16 years of age undergoing an elective procedure under GA were included. The anaesthetist for each list completed a case report form for each patient, which was anonymised and uploaded to the electronic database. Each centre also completed a questionnaire about their delivery of P‐POA.

**Exclusion Criteria:** Children undergoing emergency or repeated procedures under GA were excluded from the study, as were sedation or local anaesthetic cases.

**Results:** The survey found that 80/98 centres (82%) have a P‐POA service, with 84% of these being nurse‐led. Out of 7286 cases recorded, 4390 patients (59%) had P‐POA, most commonly through telephone appointments. Among those pre‐assessed, 76% (3375 cases) did not require anaesthetic consultant involvement, with 49% (503 cases) resolved with notes review only. In children seen in P‐POA, 15% had management altered, most commonly for clinical decisions such as bed allocation. There was no significant difference in on‐the‐day cancellations between children who had and had not been seen in P‐POA, with the cancellation rate for each being ~4%. Without P‐POA, a child has a 9% chance of a change on the day (i.e. not proceeding as planned), which reduces to 7% with POA (*p* < 0.05). Anxiety was an issue preoperatively in 17% of children without P‐POA, which reduces to 12% with P‐POA (*p* < 0.05).

**Discussion and Conclusion:** This study indicates that most UK hospitals offer paediatric pre‐operative assessment, which likely improves theatre efficiency, patient experience, and reduces perioperative anxiety. Further statistical analysis of these data is ongoing to better understand the full impact of P‐POA on paediatric surgical outcomes.

**References**

1. APAGBI Best Practice Pre‐assessment Standards in Children. Association of Paediatric Anaesthetists of Great Britain and Ireland. https://www.apagbi.org.uk/sites/default/files/2022‐05/Best%20Practice_Pre‐assessment%20standards%20in%20Children%20%202022%20‐%20Published.pdf. Published May 2022. Accessed January 30, 2024.
2. Royal College of Anaesthetists. Guidelines for the Provision of Anaesthetic Services (GPAS) Chapter 10: Guidelines for the Provision of Paediatric Anaesthesia Services 2024. Royal College of Anaesthetists. https://www.rcoa.ac.uk/gpas/chapter‐10. Published January 31 2023. Accessed January 30, 2024.

## The effects of dexmedetomidine on intraoperative neurophysiologic monitoring modalities during corrective scoliosis surgery in pediatric patients: A systematic review

### Ahmed Abdelaal Ahmed Mahmoud Metwally Alkhatip^1,2^; Kerry Elizabeth Mills^3^; Olivia Hogue^4^; Amr Sallam^5^; Mohamed Khaled Hamza^6^; Ehab Farag^2^; Hany Mahmoud Yassin^7^; Mohamed Wagih^6^; Ahmed Mohamed Ibrahim Ahmed^8^; Mohamed Hussein Helmy^6^; Mohamed Elayashy^6^

#### ^1^Department of Anaesthesia, Birmingham Children's Hospital, Birmingham, UK; ^2^Department of Anaesthesia, Faculty of Medicine, Beni Suef University, Beni Suef, Egypt; ^3^Faculty of Science and Technology, University of Canberra, Canberra, Australia; ^4^Department of Quantitative Health Sciences, Cleveland Clinic, Cleveland, Ohio, USA; ^5^Department of Anaesthesia, Tallaght University Hospital, Dublin, Ireland; ^6^Department of Anaesthesia, Faculty of Medicine, Cairo University, Cairo, Egypt; ^7^Department of Anaesthesia, Faculty of Medicine, Fayoum University, Fayoum, Egypt; ^8^Department of Anaesthesia, Addenbrooke Hospital, Cambridge University Hospitals, Cambridge, UK

Here is the updated citation:

This abstract has recently been published and can be found at the following DOI: Ahmed Abdelaal A, Mahmoud Metwally A, et al. The effects of dexmedetomidine on intraoperative neurophysiologic monitoring modalities during corrective scoliosis surgery in pediatric patients: a systematic review. *Paediatr Anaesth*. 2024 Feb;34(2):112‐120. doi: 10.1111/pan.14795. [PMID: 37927199]

## Risk of dying in paediatric anaesthesia: Evaluation, discussion, and consent

### Tom C. Lyne^1^; Harry D. Everton^2^; Lisa Barkley^3^; Benjamin J. Blaise^4,5^

#### ^1^Barnet Hospital, Royal Free London NHS Foundation Trust, Barnet, UK; ^2^Whittington Hospital, Whittington Foundation NHS Trust, London, UK; ^3^Paediatric Psychology, Evelina London Children's Hospital, Guy's and St Thomas' NHS Foundation Trust, London, UK; ^4^Department of Paediatric Anaesthetics, Evelina London Children's Hospital, Guy's and St Thomas' NHS Foundation Trust, London, UK; ^5^Centre for the Developing Brain, School of Biomedical Engineering and Imaging Sciences, King's College London, St. Thomas' Hospital, London, UK

This abstract has recently been published as correspondence in the BJA and can be found at the following DOI: Lyne TC, Everton HD, Barkley L, Blaise BJ. Limitations of the consent process in paediatric anaesthesia: the Death during Anaesthesia Risk and Explanation (DARE) audit. *Br J Anaesth*. 2024 Feb;132(2):150‐158. doi: 10.1016/j.bja.2023.10.012. Epub 2023 Dec 1.

